# Waist circumference does not improve established cardiovascular disease risk prediction modeling

**DOI:** 10.1371/journal.pone.0240214

**Published:** 2020-10-02

**Authors:** Matthew W. Nelms, Andrew G. Day, Xuemei Sui, Steven N. Blair, Robert Ross

**Affiliations:** 1 School of Kinesiology and Health Studies, Queen’s University, Kingston, Ontario, Canada; 2 Kingston Health Sciences Centre, Kingston General Health Research Institute, Kingston, Ontario, Canada; 3 Department of Exercise Science, University of South Carolina, Columbia, South Carolina, United States of America; 4 Division of Endocrinology and Metabolism, School of Medicine, Queen’s University, Kingston, Ontario, Canada; Karolinska Institutet, SWEDEN

## Abstract

Despite considerable evidence demonstrating that waist circumference (WC) is independently associated with cardiovascular disease (CVD) and/or all-cause mortality, whether the addition of WC improves risk prediction models is unclear. The objective was to evaluate the improvement in risk prediction with the addition of WC, alone or in combination with BMI, to the Framingham Risk Score (FRS) and a population specific model. 34,377 men and 9,477 women aged 20 to 79 years who completed a baseline examination at the Cooper Clinic (Dallas, TX) during 1977–2003 and enrolled in the Aerobics Center Longitudinal Study (ACLS). WC was measured at the level of the umbilicus and expressed as a continuous variable. Deaths among participants were identified using the National Center for Health Statistics National Death Index. A total of 728 fatal cardiovascular disease (CVD) events occurred over a mean follow-up period of 13.1 ± 7.5 years. WC was significantly higher in CVD decedents (*P* = .002). The FRS C-statistic for fatal CVD in men was 0.836 (0.816–0.855) and 0.883 (0.851–0.915) in women. The addition of WC did not improve the C-statistic in men (0.831 (0.809–0.853)) or women (0.883 (0.850–0.916)). Similar findings were observed for non-fatal CVD and all-cause mortality, and when WC was added to a population specific model. Upon adding WC, the net-reclassification index was 0.024 with an integrated discrimination improvement of -0.0004. The addition of WC, alone or in combination with BMI, did not substantively improve risk prediction for CVD or all-cause mortality compared to the Framingham Risk Score or a population specific model.

## Introduction

Considerable evidence has established that waist circumference (WC) is independently associated with cardiovascular disease (CVD) [1–3] and all-cause mortality [1, 4–6], with the full strength of the association realized only upon adjustment for BMI [5, 7, 8]. Despite this evidence, the measurement of WC is not routinely obtained in clinical practice [9–11]. One plausible explanation for the resistance to measure WC is the lack of evidence evaluating whether the addition of WC improves established risk prediction models [12].

According to the American Heart Association, novel risk markers must at least demonstrate an independent association with health risk after adjusting for established risk factors [13]. Indeed, it is well established that WC alone or in combination with BMI is associated with morbidity and mortality independent of commonly obtained risk factors [1, 8, 14]. However, this observation alone is insufficient as many biomarkers meet this criterion but fail to improve risk prediction. Thus, several more stringent statistical measures including discrimination, calibration and reclassification have been devised to provide a global evaluation of risk prediction with the novel biomarker, as no one measure provides a comprehensive assessment [13, 15].

We are aware of only one study that has evaluated whether the addition of WC improves risk prediction using several measures of model performance. The Emerging Risk Factors Collaboration evaluated a population specific CVD risk model containing conventional risk factors in ~145,000 adults and assessed whether the addition of WC would improve risk prediction [14]. Although WC was independently associated with CVD events, there was no meaningful improvement in risk prediction.

The failure of a marker to improve risk prediction is not unexpected. Cook and others, have demonstrated the challenge for any biomarker to considerably improve prognostic performance [15–19]. Pencina et al., estimate that age, sex and ethnicity account for 63–80% of the prognostic value of CVD risk prediction models [20]. Furthermore, WC may fail to improve the prognostic performance of risk prediction models, because its effect on CVD risk may be mediated through its effect on downstream cardiometabolic risk factors which are already included in the prediction models [21, 22].

In this study we investigated the incremental risk prediction improvement of adding WC, alone or in combination with BMI, to the Framingham Risk Score and a population specific model. Furthermore, we assessed the association between the change in WC and outcomes, with investigation of risk prediction improvement.

## Methods

### Study population

Participants were 34,377 men and 9,477 women aged 20 to 79 years who completed a baseline examination at the Cooper Clinic (Dallas, TX) during 1977–2003 and voluntarily enrolled in the Aerobics Center Longitudinal Study (ACLS). The study population is predominantly white, well-educated, United States residents from all 50 states and from middle to upper socioeconomic stratum [23]. At baseline, participants were free of known CVD and cancer, and had normal resting electrocardiograms. All participants were aware of the purpose of the study and provided written consent prior to study participation. The ACLS protocol was subject to annual review by the Cooper Institute’s Institutional review board.

### Clinical examination

Patients underwent a full preventive medical examination including assessment by a physician, fasted blood chemistry, personal and family health history, anthropometry, resting blood pressure measurement. All examinations were conducted by trained technicians following standardized measurement protocols [24].

WC was measured at the level of the umbilicus to the nearest 0.5 cm [24]. Cigarette smoking (current smoker or not), was obtained from a standardized questionnaire. Blood pressure was measured using standard auscultatory methods. Serum samples were analyzed for lipids and glucose using standardized automated bioassays [24]. Presence of diabetes was defined as fasting glucose >126 mg/dL (7.0 mmol/L), previous physician diagnosis of diabetes, or reported insulin use. Participants with self-reported high blood pressure were categorized as receiving treatment systolic blood pressure. Height and weight were measured using a stadiometer and a physician’s scale. BMI was calculated according to the formula: Mass (kg) / Height (m^2^).

### Assessment of outcomes

Fatal CVD and all-cause mortality were ascertained through linking the ACLS cohort with the National Center for Health Statistic’s National Death Index. Follow-up occurred from the date of baseline examination until the date of death, or December 31, 2003 for survivors. Participants who passed away with less than one year of follow-up were excluded. CVD death was identified using the International Classification of Diseases, Ninth Revision codes 390.0 to 458.9 or equivalent from the Tenth Revision for CVD [25]. Non-fatal CVD events were ascertained from responses to mail-back health surveys in 1982, 1986, 1990, 1995, 1999 and 2004. The aggregate survey response rate across all survey periods in the ACLS is ≈ 65 percent [26]. Non-fatal CVD endpoints were defined as a physician diagnosis of myocardial infarction or stroke or a coronary revascularization procedure (coronary artery bypass graft or percutaneous coronary intervention). In participants reporting multiple non-fatal CVD events, the first event was used in the analysis. Participants with incomplete data were not included in the analysis.

### Application of the Framingham Risk Score

The General Framingham Risk Score (FRS) published by D’Agostino et al, was calculated for all participants [27]. A population specific risk model (PSM) was generated for each participant using the same variables as the FRS, including age, sex, systolic blood pressure, total cholesterol, high-density lipoprotein cholesterol (HDL), and dichotomous variables for treated high blood pressure, smoking and diabetes mellitus [27]. CVD risk was denoted using the following categories <5%, 5% to <10%, 10% to <20% and ≥20% [14].

### Statistical analysis

Baseline characteristics were compared within sex between CVD survivors and decedents. Continuous variables were described by means and standard deviations and compared by Welch’s two-sample t-test. Binary variables (smoking and diabetes) were described as counts and percentages and compared between groups by the Chi-Squared test.

Separate Cox proportional hazard models were used to model: 1) time to fatal CVD, 2) non-fatal CVD and 3) all-cause mortality. The hazard ratios (HRs) with 95% confidence intervals (CIs) were estimated per 1SD increase in baseline value for WC and BMI. Separate sex stratified models were estimated to adjust for: 1) age and smoking; 2) FRS; 3) FRS plus WC and BMI; 4) a population specific model (PSM) with variables as described above; 5) PSM model plus WC and BMI.

In a subset of the population with serial measures, separate Cox proportional hazard models were used to model: 1) time to fatal CVD, 2) non-fatal CVD and 3) all-cause mortality. Men with at least 6 months between measures and at least one year of follow-up after the second visit were included. The HRs with 95% CIs were estimated per 1 unit increase in baseline value for WC (cm) and BMI (kg/m^2^) and 1 unit of change for WC and BMI. Separate models were estimated to adjust for baseline and change values: 1) age and smoking; 2) FRS score; 3) PSM model; and 4) PSM model plus WC plus BMI.

Uno’s C-statistic was used to measure model discrimination in censored time-to-event data of the Cox proportional hazard models [28]. CIs were ascertained for the C-statistic using 50 perturbation samples.

Reclassification analysis requires binary outcomes with a predetermined time interval, thus 10-year binary logistic regression models among men who enrolled on or before December 31, 1993 were fit [25]. Due to the low number of events among females during the 10-year follow-up, they were removed from the analyses. The net-reclassification index (NRI) [29] and the integrated discrimination improvement (IDI) [17] were calculated using previously suggested threshold values for 10-year CVD risk categories (<5%, 5% to <10%, 10% to <20% and ≤20%) [14, 17]. The continuous NRI (C-NRI) which uses no categories was also assessed. The relative IDI was calculated as the IDI divided by the discrimination slope of the base model [30]. The C-statistic was calculated as a measure of model discrimination. The Hosmer-Lemeshow test was used to assess model calibration.

A canonical correlation was computed to measure the association between WC and the collection of cardiometabolic risk factors (total cholesterol, HDL, systolic blood pressure, triglycerides and fasting blood glucose). The same analysis was repeated using change values for the variables above.

All statistical analyses were performed using SAS for Windows (9.4; SAS Institute, Inc., Cary, NC). Two-sided p values and 95% CIs are presented.

## Results

There were 728 fatal CVD events among 43,854 individuals followed for an average of 13.1 ± 7.5 years with a range of 1 to 26.4 years, for a total of 573,961 person-years of observation. Table 1 displays the baseline characteristics of the total population according to sex and CVD mortality status. Mean age at baseline was 45.5 ± 10.9 years for women and 44.9 ± 9.9 years for men. In both men and women, decedents were older, had higher total cholesterol, lower HDL, higher triglycerides, higher blood pressure and higher WC (*P* < .002). Decedents were more likely to self-report high blood pressure and to have diabetes (*P <* .003). In men only, decedents had higher BMI and were more likely to smoke (*P* < .002).

**Table 1:** Baseline characteristics of study population according to CVD mortality and sex.

	Women		Men	
	Survivors n = 9,394	Decedents n = 83	Survivors n = 33,732	Decedents n = 645
Age, mean (SD), y	45.4 (10.8)	61.1 (11.3) *	44.6 (9.7)	55.7 (10.5) *
Total Cholesterol, mean (SD), mmol/L	5.2 (0.9)	5.9 (1.1) *	5.4 (1.0)	5.8 (1.0) *
HDL Cholesterol, mean (SD), mmol/L	1.6 (0.4)	1.5 (0.4) *	1.2 (0.3)	1.1 (0.3) *
Triglycerides, mean (SD), mmol/L	1.1 (0.7)	1.5 (0.9) *	1.5 (1.1)	1.8 (1.2) *
Systolic BP, mean (SD), mm Hg	114.3 (15.1)	128.2 (20.3) *	121.2 (13.3)	129.8 (16.6) *
Treated systolic BP^a^, n (%)	1,060 (11.3)	18 (21.7) *	4,896 (14.5)	211 (32.7) *
Smoking, n (%)	829 (8.8)	7 (8.4)	5,692 (16.9)	168 (26.1) *
Diabetes^b^, n (%)	396 (4.2)	11 (13.3) *	1,548 (4.6)	84 (13.0) *
Waist circumference, mean (SD), cm	74.6 (11.1)	78.9 (11.9) *	94.0 (10.9)	97.7 (13.2) *
Body mass index, mean (SD), kg/m^2^	23.8 (4.4)	24.7 (4.2)	26.7 (3.9)	27.2 (4.2) *

BP, blood pressure; CVD, cardiovascular disease; SD, standard deviation; y, years.

^a^ Defined as self-reported high blood pressure.

^b^ Defined as a history of physician diagnosed diabetes, use of insulin, or measured fasting glucose level ≥ 7.0 mmol/L.

* Indicates a significant difference between survivors and decedents (*P* < .005).

### Performance of the Framingham Risk Score in the ACLS population

The FRS provided good discrimination as assessed by the C-statistic for fatal CVD (male 0.836, female 0.883), non-fatal CVD (male 0.734, female 0.767) and all-cause mortality (male 0.764, female 0.805). The PSM demonstrated good discrimination as assessed by the C-statistic for fatal CVD (male 0.841, female 0.903), non-fatal CVD (male 0.744, female 0.787) and all-cause mortality (male 0.772, female 0.823).

### Associations between WC, change in WC and events

In sex-stratified Cox proportional hazard models adjusted for age and smoking, WC was significantly related to fatal CVD, non-fatal CVD and all-cause mortality in men, and fatal CVD and all-cause mortality in women (S1 Table). Upon adjustment for the PSM variables, the associations in men between WC and fatal CVD and all-cause mortality remained significant (*P*< .001) while the association with non-fatal CVD lost significance (*P*>.25). In women, the associations were no longer significant (*P*>.43). Further adjustment for BMI did not change the associations.

Change values were calculated in a subset of the population with serial measures. There were 217 fatal CVD events among 12,495 men followed for an average of 14.8 ± 7.1 years from their second visit. The mean time between measures was 2.4 ± 2.6 years and the mean change in WC was 0.04 ± 5.89 cm. Decedents were older, had higher total cholesterol, higher systolic blood pressure, lower HDL and a higher WC at baseline (*P* < .03). Survivors were less likely to self-report high blood pressure and to have diabetes (*P* < .001). The change in WC was not significantly different between survivors and decedents (*P*>0.73).

In Cox proportional hazard models adjusted for age, smoking and baseline WC, the change in WC was not significantly related to the outcomes of interest (S2 Table).

### WC and risk prediction: Model discrimination and calibration

Table 2 reports the changes in model discrimination with the addition of WC to the FRS and the PSM across outcomes. Upon addition of WC to the FRS in men, alone or in combination with BMI, the change in discrimination measured by the C-statistic was non-significant for fatal CVD (ΔC-statistic -0.0068–0.0000), non-fatal CVD (ΔC-statistic 0.0003–0.0011) and all-cause mortality (ΔC-statistic -0.0078–0.0001). Non-significant changes were also observed in women and with the addition of WC to the PSM (Table 2).

**Table 2:** Changes in model discrimination with the addition of WC and BMI.

	C-statistic (95% CI)	Likelihood Ratio *X*^2^	AIC	C-statistic (95% CI)	Likelihood Ratio *X*^2^	AIC
	Male			Female		
	***Fatal CVD events*** (34,377 participants, 645 events)			***Fatal CVD events*** (9,477 participants, 83 events)		
Age + smoking	0.817 (0.791–0.844)	957.3	11518.6	0.892 (0.857–0.928)	204.4	1141.8
Age, smoking + WC + BMI	0.830 (0.807–0.853)	1033.3	11446.6	0.898 (0.857–0.930)	211.0	1139.2
FRS	0.836 (0.816–0.855)	852.4	11621.6	0.883 (0.851–0.915)	160.1	1184.0
FRS + WC	0.831 (0.810–0.853)	873.4	11602.5	0.883 (0.850–0.916)	161.3	1184.9
FRS + BMI	0.836 (0.813–0.859)	860.4	11615.5	0.883 (0.852–0.914)	160.1	1186.0
FRS + WC + BMI	0.829 (0.807–0.851)	878.7	11599.2	0.883 (0.850–0.916)	163.2	1184.9
PSM	0.841 (0.822–0.860)	1167.3	11318.7	0.904 (0.870–0.938)	226.3	1129.8
PSM + WC	0.845 (0.823–0.867)	1188.9	11299.0	0.905 (0.875–0.936)	226.9	1131.2
PSM + BMI	0.844 (0.819–0.869)	1180.0	11307.9	0.904 (0.877–0.931)	226.3	1131.8
PSM + WC + BMI	0.844 (0.823–0.866)	1189.5	11300.5	0.904 (0.872–0.937)	228.3	1131.8
	***Non-fatal CVD events*** (18,918 participants, 745 events)			***Non-fatal CVD events*** (4,533 participants, 80 events)		
Age + smoking	0.728 (0.704–0.751)	543.4	13667.5	0.784 (0.732–0.835)	86.3	1201.7
Age, smoking + WC + BMI	0.728 (0.710–0.746)	551.0	13664.0	0.783 (0.733–0.833)	88.0	1204.0
FRS	0.734 (0.713–0.755)	408.3	13800.6	0.767 (0.706–0.829)	71.0	1215.0
FRS + WC	0.734 (0.717–0.752)	408.4	13802.6	0.768 (0.702–0.834)	71.0	1217.0
FRS + BMI	0.735 (0.718–0.753)	408.5	13802.4	0.769 (0.716–0.823)	71.6	1216.4
FRS + WC + BMI	0.735 (0.718–0.752)	408.7	13804.2	0.770 (0.722–0.818)	72.5	1217.5
PSM	0.744 (0.724–0.764)	645.3	13575.7	0.787 (0.736–0.838)	95.2	1202.8
PSM + WC	0.744 (0.722–0.764)	646.6	13576.3	0.787 (0.734–0.841)	95.3	1204.7
PSM + BMI	0.744 (0.724–0.764)	645.9	13577.1	0.788 (0.733–0.842)	95.3	1204.7
PSM + WC + BMI	0.745 (0.720–0.769)	646.8	13578.2	0.788 (0.729–0.846)	96.1	1205.9
	***All-cause mortality*** (34,377 participants, 1,823 events)			***All-cause mortality*** (9,477 participants, 288 events)		
Age + smoking	0.763 (0.747–0.779)	1787.4	33473.3	0.819 (0.789–0.848)	427.4	4264.1
Age, smoking + WC + BMI	0.768 (0.746–0.789)	1849.1	33415.7	0.822 (0.787–0.856)	435.6	4259.8
FRS	0.760 (0.733–0.788)	1443.7	33815.1	0.801 (0.771–0.831)	309.6	4379.9
FRS + WC	0.758 (0.739–0.776)	1454.1	33806.7	0.800 (0.769–0.831)	311.0	4380.4
FRS + BMI	0.761 (0.737–0.784)	1444.8	33816.0	0.800 (0.775–0.825)	309.9	4381.6
FRS + WC + BMI	0.753 (0.726–0.780)	1466.2	33796.6	0.800 (0.767–0.833)	311.8	4381.7
PSM	0.772 (0.756–0.789)	1982.3	33288.5	0.823 (0.795–0.851)	451.6	4249.9
PSM + WC	0.773 (0.753–0.793)	1998.1	33274.7	0.824 (0.792–0.856)	453.8	4249.6
PSM + BMI	0.773 (0.752–0.794)	1990.7	33282.1	0.823 (0.791–0.855)	453.1	4250.3
PSM + WC + BMI	0.773 (0.750–0.795)	1998.9	33275.9	0.824 (0.790–0.858)	453.8	4251.6

Analyses were restricted to participants with complete information on all adjusted variables. Participants included within the Any CVD and Non-fatal CVD must have completed at least one mail-in survey. FRS (Framingham Risk Score) applied to study population. PSM (population specific model) = age, sex, systolic blood pressure, treated systolic blood pressure, total cholesterol, HDL cholesterol, smoking, diabetes. BMI, body mass index; WC, waist circumference; AIC, Akaike information criterion.

In a subset of the population with serial measures, the addition of the change in WC to the PSM model was examined (Table 3). The C-statistic for the PSM in fatal CVD was 0.818 (0.781–0.854) and did not change significantly with the inclusion of change values [0.826 (0.777–0.876)]. The addition of WC to the model did not significantly improve the model discrimination (Table 3). Similar results were observed for non-fatal CVD and all-cause mortality.

**Table 3:** Model discrimination with addition of WC and change in WC in men: Time-to-event analysis.

	C-statistic (95% CI)	Δ C-statistic	Likelihood Ratio *X*^2^	AIC
***Fatal CVD events*** (12,495 participants, 217 events)				
PSM	0.818 (0.781–0.854)	\	285.0	3470.2
PSM + WC	0.820 (0.785–0.855)	0.002	291.7	3465.4
PSM + BMI	0.821 (0.789–0.854)	0.003	290.0	3467.1
PSM + WC + BMI	0.820 (0.779–0.862)	0.002	291.8	3467.4
Change PSM	0.826 (0.777–0.876)	0.009	304.6	3464.6
Change PSM + WC	0.830 (0.789–0.869)	0.012	312.0	3462.1
Change PSM + BMI	0.830 (0.778–0.882)	0.012	310.7	3462.4
Change PSM + WC + BMI	0.829 (0.782–0.871)	0.011	312.8	3464.3
***Non-fatal CVD events*** (9,323 participants, 402 events)				
PSM	0.741 (0.665–0.817)	\	287.9	6022.1
PSM + WC	0.743 (0.671–0.815)	0.002	291.5	6020.5
PSM + BMI	0.743 (0.685–0.800)	0.002	290.8	6021.1
PSM + WC + BMI	0.743 (0.664–0.822)	0.002	291.5	6022.4
Change PSM	0.754 (0.665–0.842)	0.013	410.0	5914.0
Change PSM + WC	0.753 (0.654–0.8523	0.012	415.6	5912.3
Change PSM + BMI	0.754 (0.667–0.842)	0.013	413.7	5914.2
Change PSM + WC + BMI	0.752 (0.65890.845)	0.011	416.2	5915.8
***All-cause mortality*** (12,495 participants, 688 events)				
PSM	0.748 (0.715–0.780)	\	619.9	11324.0
PSM + WC	0.748 (0.707–0.789)	0.000	634.2	11323.7
PSM + BMI	0.749 (0.716–0.782)	0.001	634.1	11323.9
PSM + WC + BMI	0.749 (0.711–0.788)	0.001	634.4	11325.6
Change PSM	0.748 (0.716–0.781)	0.000	654.4	11315.5
Change PSM + WC	0.748 (0.715–0.781)	0.000	656.6	11317.4
Change PSM + BMI	0.750 (0.706–0.793)	0.002	657.9	11316.0
Change PSM + WC + BMI	0.750 (0.710–0.790)	0.002	660.6	11317.3

Analyses were restricted to participants with complete information on all adjusted variables. FRS (Framingham Risk Score) as published by D’Agostino et al., 2008 [27]. PSM (population specific model) = age, sex, systolic blood pressure, treated systolic blood pressure, total cholesterol, HDL cholesterol, smoking, diabetes. BMI, body mass index; WC, waist circumference; AIC, Akaike information criterion.

In men with at least 10 years of observation, the addition of WC to the FRS or the PSM resulted in non-significant changes in model discrimination (S3 Table). The PSM model C-statistic for fatal CVD was 0.842 (0.816–0.868). The addition of WC to the model produced a C-statistic of 0.843 (0.817–0.869). The Hosmer-Lemeshow goodness-of-fit test was used to measure model calibration. Across all outcomes, the FRS model demonstrated poor calibration, which persisted with the addition of WC to the model. Calibration in the PSM model was good for fatal-CVD and poor for non-fatal and all-cause mortality.

### WC and risk prediction: Reclassification indices

Men with at least 10 years of follow-up were included (n = 22,915). The addition of WC to the PSM resulted in minimal net-reclassification (NRI) for fatal CVD (-1.6% to -0.6%), non-fatal CVD (-1.1% to 0.4%) and all-cause mortality (-0.7% to 0.0%) (Table 4). A descriptive approach of the reclassification analyses was employed due to the small reclassification observed with the addition of WC and thus the associated clinical relevance was marginal. Similar reclassification was observed when risk threshold values of 0–6%, 6–20% and >20% were used. Generally, the addition of WC to the FRS and PSM model resulted in minimal reclassification of cases and controls.

**Table 4:** Reclassification of 10-year risk with the addition of WC and BMI in men.

	NRI			Continuous NRI	IDI	rIDI
	Cases	Controls	Net-change			
***Fatal CVD*** (22,915 participants, 198 events)						
FRS + WC	0.025	-0.001	0.024	0.17	-0.0004	-0.96
FRS + BMI	0.020	-0.001	0.019	0.09	-0.0004	-1.12
FRS + WC + BMI	0.025	-0.001	0.024	0.17	0.0001	0.30
PSM + WC	-0.015	-0.001	-0.016	0.21	0.0002	0.44
PSM + BMI	-0.015	-0.001	-0.016	0.27	-0.0002	-0.49
PSM + WC + BMI	-0.005	-0.001	-0.006	0.22	0.0005	1.32
***Non-fatal CVD*** (14,885 participants, 372 events)						
FRS + WC	-0.003	0.000	-0.003	0.08	0.0000	-0.07
FRS + BMI	-0.003	0.000	-0.003	0.12	0.0000	-0.02
FRS + WC + BMI	0.000	0.020	0.020	0.02	0.0001	0.45
PSM + WC	-0.011	0.000	-0.011	-0.07	0.0001	0.31
PSM + BMI	0.000	0.000	0.000	0.08	0.0000	0.00
PSM + WC + BMI	0.003	0.002	0.004	0.01	0.0005	1.32
***All-cause mortality*** (22,915 participants, 534 events)						
FRS + WC	0.000	-0.001	-0.001	0.04	-0.0001	-0.15
FRS + BMI	-0.004	0.000	-0.004	-0.01	0.0001	0.15
FRS + WC + BMI	0.004	-0.001	0.003	0.16	0.0008	1.70
PSM + WC	-0.006	-0.001	-0.007	0.12	0.0003	5.52
PSM + BMI	0.000	0.000	0.000	0.10	0.0000	-0.02
PSM + WC + BMI	-0.004	0.000	-0.004	0.09	0.0007	1.50

Analyses were restricted to male participants who could have been followed for at least 10 years and those with complete information on all adjusted variables. FRS (Framingham Risk Score) applied to the study population. PSM (population specific model) BMI, body mass index; WC, waist circumference; NRI, net-reclassification index; IDI, integrated discrimination improvement; rIDI, relative integrated discrimination improvement. FRS enhanced models are compared to the base FRS, PSM enhanced models are compared to the base PSM.

However, with the addition of WC to the PSM, the C-NRI reported substantial positive reclassification for fatal CVD (21.0 to 21.7%), with similar observations upon addition to the FRS. In contrast, the C-NRI was attenuated for all-cause mortality (8.5% to 11.8%), and non-fatal CVD (-6% to 1.3%). Although the C-NRI reports model improvement, the integrated discrimination improvement (IDI), which provides information on the magnitude of risk change, was small across outcomes (fatal CVD 0.0002–0.0005, non-fatal CVD 0.0001–0.0005, all-cause mortality 0.0003–0.0007). Similarly, the relative IDI (rIDI) demonstrated minimal change in model discrimination for fatal CVD (0.4–1.3), non-fatal CVD (0.3–1.3) and all cause mortality (1.5–5.5).

### Association between WC and intermediate risk factors

The results here suggest that addition of WC to the FRS and PSM does not substantively improve risk prediction. We posited that this is partially explained by the association between WC and several cardiometabolic risk factors (total cholesterol, HDL, systolic blood pressure, triglycerides and fasting blood glucose). Indeed, the canonical correlation demonstrated that WC explained 36% of the variance in the cardiometabolic risk factors, and 19% of the variance after adjustment for age and sex.

Similar to the association described above, a canonical correlation demonstrated that change in WC explained 11% of the variance in the change in the cardiometabolic risk factors, and 10% of the variance after adjustment for age.

## Discussion

The primary finding of this study is that the addition of WC did not substantively improve the performance of the Framingham Risk Score or a population specific model for predicting fatal CVD, non-fatal CVD and all-cause mortality. This observation remained consistent across both traditional measures of model performance (C-statistic, Likelihood ratio test) and more novel measures (NRI, IDI). However, the C-NRI demonstrated improved net-reclassification for fatal CVD.

The Emerging Risk Factors Collaboration published the only other study evaluating the addition of WC, alone or in combination with BMI, to a CVD risk prediction model using several measures of model performance. Similar to our findings, they observed a significant association between WC and CVD independent of traditional risk factors [14]. Nonetheless, they observed no improvement in model discrimination (ΔC-statistic -0.0001) or reclassification (NRI -0.05%, IDI 0.0004) with the addition of WC to their population specific model. The current study extends this work by evaluating the performance of the FRS in the study population and the effect of adding WC to this established model. Moreover, we considered additional measures of model performance, the C-NRI and the rIDI, to evaluate the addition of WC to an established risk model.

The C-NRI evaluates the change in reclassification without the use of pre-specified risk thresholds [30]. The addition of WC to the FRS and PSM resulted in a sizeable reclassification for fatal CVD as measured by the C-NRI. Improvement in the C-NRI suggests that with the addition of WC to the FRS, individuals who experience an event have a higher predicted risk compared to the FRS alone. However, the clinical importance of changes in C-NRI are not fully understood [30]. The IDI is a complementary statistic that is used to help quantify the magnitude of the change in risk. Moreover, the rIDI is a ratio of the IDI and the base model discrimination slope, quantifying the relative change in discrimination between cases and controls [30]. Across outcomes in our study the rIDI was relatively small suggesting minimal changes in the FRS’s and PSM’s ability to separate cases and controls with the addition of WC.

There are likely several factors contributing to the failure of WC to improve risk prediction. The most prominent is that non-modifiable risk factors including age, sex and ethnicity account for much of the prognostic performance [20]. For example, Pencina et al., observed that the addition of systolic blood pressure, non-HDL-cholesterol, diabetes or smoking to a model including, age, sex and ethnicity improved the C-statistic for CVD by only 0.004–0.013 [20]. We observed similar changes in the C-statistic with the addition of these traditional risk factors and observed that age and sex alone accounted for 90–93% of the PSM performance. Moreover, the distribution of the risk factor of interest between cases and controls must be sufficiently separated to effectively discriminate between the groups with a high sensitivity and specificity, which is rarely the case [31]. Therefore, both traditional risk factors and novel biomarkers face substantial challenges in improving risk prediction models. Moreover, any additive value of WC is likely overwhelmed by more proximal, causative cardiometabolic risk factors, such as abnormal blood glucose and elevated blood pressure. In this study we confirm that WC is associated with metabolic health risk [21, 22], and that change in WC is associated with a corresponding change in the collection of cardiometabolic risk factors. It is well established that WC is associated with visceral adipose tissue (VAT) [12]. It is also known that VAT is a strong predictor of cardiometabolic risk [32–34], and may be a central mechanism by which an elevated WC contributes to an increased risk of morbidity and mortality [35–39]. The reader is refereed to the review by Neeland et al [39] for a detailed review of the literature investigating the associations between VAT and cardiometabolic risk.

Evaluating the clinical importance of a novel biomarker solely by its ability to improve risk prediction may be short sighted. Although the addition of new biomarkers results in limited improvement in risk prediction beyond non-modifiable factors, the improvement of cardiometabolic risk factors significantly decreases the risk of CVD [20]. For example, it is well established that a negative energy balance induced by diet and/or exercise is associated with corresponding improvements in cardiometabolic health risk in a dose-response manner [12, 40–43]. This observation is consistent with our finding demonstrating that change in WC is associated with a corresponding change in the collection of cardiometabolic risk factors. Thus, WC remains a simple evidence-based target for clinical practice, providing the clinician with an opportunity to counsel patients on the health benefits of lifestyle-based strategies designed to reduce abdominal obesity and consequently, health risk. In summary, although WC may not improve risk prediction modeling, it serves as an important modifiable treatment target for risk reduction [12].

In the current study WC was measured at the level of the umbilicus. This may have introduced error in measurement as protocols that make use of bony landmarks to measure WC offer improved reliability [44]. The WHO protocol (the midpoint between the lower border of the rib cage and the iliac crest) [45] and the NIH protocol (the superior border of the iliac crest) [46] are the preferred measures. Whether WC would have improved the FRS model had it been measured using an established method is unknown. A recent review outlines the most appropriate WC thresholds and provides rationale for thresholds that differ across ethnicity [12].

In both men and women, CVD decedents had a significantly higher WC compared to survivors, a consistent observation in the literature [1–3]. However, only in men did decedents have a significantly higher BMI compared to survivors whereas the association did not reach significance in women. This observation underscores the value of assessing WC in clinical practice, as abdominal obesity is associated with health risk regardless of BMI [12].

Strengths of the current study include a large, well described cohort [23, 26, 47] with sizable follow-up, providing a large number of events. Both anthropometric and metabolic variables were collected using standardized techniques, reducing measurement error and strengthening the validity of our observations [26]. Furthermore, the exclusion of individuals with pre-existing CVD or cancer at baseline and those with less than one year of follow-up decreased the likelihood of other factors influencing our findings. The ability to eliminate these individuals from the analyses provides increased confidence in the findings by reducing the possibility of reverse causality. The use of continuous analyses as opposed to categorical analyses strengthens the study design by reducing information loss [48, 49].

The cohort is comprised primarily of White individuals who are well educated and from middle to upper socioeconomic stratum. While the sociodemographic homogeneity of the sample may limit the generalizability (external validity), it enhances the internal validity of our findings by reducing possible confounding from these factors. There was incomplete information on medication use in our population which may have contributed to confounding. In an effort to address this limitation, we assumed that all individuals with self-reported high blood pressure at baseline were taking medication to help control their blood pressure. However, it is unlikely that the primary findings would change substantially with inclusion of such information. There were relatively few events during the first 10 years of follow-up in women with serial measures, thus they were not included in these analyses. Generalization of these findings to other populations merits appropriate caution. However, there were no substantial differences between men and women in the time-to-event analyses and thus it is unlikely there would be major differences in the 10-year findings. The dataset does not include information regarding menopausal status which is associated with the distribution of adipose tissue [50]. Whether menopausal status would have influenced the observations is unknown. The mail-in survey response rate for non-fatal CVD events was ~65%. Nonresponse bias has been previously investigated in the ACLS dataset, wherein no difference in baseline health was observed between those individuals who responded to mail in surveys and those who did not [51].

In conclusion, the addition of WC, alone or in combination with BMI, did not substantively improve risk prediction in this large sample of middle-aged, primarily White individuals. However, WC was associated with fatal CVD and all-cause mortality independent of traditional cardiometabolic risk factors. Moreover, WC was significantly associated with the collection of the cardiometabolic risk factors included within the Framingham Risk Score and a change in WC was associated with a change in these risk factors.

## Supporting information

S1 Table Associations between WC and BMI with CVD and all-cause mortality: Time-to-event analysis. (DOCX)

S2 Table Associations between WC, ΔWC, BMI and ΔBMI with events in men: Time-to-event analysis. (DOCX)

S3 Table Changes in model performance with the addition of WC and BMI: 10-year data. (DOCX)
