# Supplementary material for: Waist circumference does not improve established cardiovascular disease risk prediction modeling
Source: PLoS One. 2020 Oct 2;15(10):e0240214. doi: 10.1371/journal.pone.0240214 (PMC7531816; doi:10.1371/journal.pone.0240214)
Supplement: S1 Table — (DOCX) [file pone.0240214.s001.docx]

| **S1 Table. Associations between WC and BMI with CVD and all-cause mortality: Time-to-event analysis** | | | |
| --- | --- | --- | --- |
|  | **HR** *(95% CI)* | | |
|  | Age, smoking & sex | PSM+ | PSM++ |
| **Women** | | | |
| ***Fatal CVD events*** (9,477 participants, 83 events) | | | |
| WC | 1.30 (1.07-1.58) | 1.09 (0.88-1.36) | 1.35 (0.90-2.00) |
| BMI | 1.21 (0.98-1.50) | 1.00 (0.80-1.26) | 0.78 (0.51-1.18) |
| ***Non-fatal CVD events*** (4,533 participants, 80 events) | | | |
| WC | 1.15 (0.90-1.46) | 1.04 (0.80-1.36) | 1.21 (0.80-1.82) |
| BMI | 1.07 (0.82-1.39) | 0.95 (0.71-1.27) | 0.81 (0.51-1.28) |
| ***All-cause mortality*** (9,477 participants, 288 events) | | | |
| WC | 1.19 (1.06-1.34) | 1.11 (0.97-1.26) | 1.10 (0.88-1.38) |
| BMI | 1.18 (1.05-1.32) | 1.09 (0.96-1.24) | 1.00 (0.80-1.27) |
| **Men** |  |  |  |
| ***Fatal CVD events*** (34,377 participants, 645 events) | | | |
| WC | 1.39 (1.30-1.49) | 1.21 (1.12-1.31) | 1.27 (1.10-1.47) |
| BMI | 1.37 (1.28-1.48) | 1.16 (1.07-1.26) | 0.94 (0.81-1.10) |
| ***Non-fatal CVD events*** (18,918 participants, 745 events) | | | |
| WC | 1.10 (1.02-1.18) | 0.95 (0.88-1.03) | 0.93 (0.81-1.08) |
| BMI | 1.12 (1.04-1.21) | 0.97 (0.89-1.06) | 1.03 (0.88-1.20) |
| ***All-cause mortality*** (34,377 participants, 1,823 events) | | | |
| WC | 1.21 (1.16-1.27) | 1.11 (1.05-1.17) | 1.15 (1.05-1.27) |
| BMI | 1.19 (1.14-1.25) | 1.08-1.03-1.14) | 0.96 (0.87-1.06) |
| Analyses were restricted to participants with complete information on all adjusted variables. HRs are presented per 1 SD of baseline value: 4.4 kg/m^2^ and 3.9 kg/m^2^ higher BMI, and 11.1 cm and 11.0 cm higher WC for women and men, respectively. PSM (population specific model) = age, sex, systolic blood pressure, treated systolic blood pressure, total cholesterol, HDL cholesterol, smoking, diabetes. BMI, body mass index; CVD, cardiovascular disease; WC, waist circumference.  + indicates the inclusion of either WC or BMI in the model  ++ indicates the inclusion of both WC and BMI in the model | | | |
