# Supplementary material for: Waist circumference does not improve established cardiovascular disease risk prediction modeling
Source: PLoS One. 2020 Oct 2;15(10):e0240214. doi: 10.1371/journal.pone.0240214 (PMC7531816; doi:10.1371/journal.pone.0240214)
Supplement: S2 Table — (DOCX) [file pone.0240214.s002.docx]

| **S2 Table. Associations between WC, ∆WC, BMI and ∆BMI with events in men: Time-to-event analysis** | | | |
| --- | --- | --- | --- |
|  | **HR** *(95% CI)* | | |
|  | Age, smoking & sex | ∆PSM+ | ∆PSM++ |
| ***Fatal CVD events*** (12,495 participants, 217 events) | | | |
| WC | 1.03 (1.02-1.05) | 1.02 (1.00-1.04) | 1.01 (0.98-1.04) |
| ∆WC | 1.01 (0.98-1.03) | 1.00 (0.97-1.02) | 0.99 (0.96-1.020 |
| BMI | 1.10 (1.05-1.14) | 1.06 (1.01-1.10) | 1.03 (0.94-1.12) |
| ∆BMI | 1.05 (0.94-1.16) | 1.03 (0.92-1.16) | 1.06 (0.93-1.22) |
| ***Non-fatal CVD events*** (9,323 participants, 402 events) | | | |
| WC | 1.02 (1.01-1.03) | 1.01 (1.00-1.02) | 1.00 (0.98-1.03) |
| ∆WC | 0.99 (0.98-1.01) | 0.99 (0.97-1.01) | 0.98 (0.96-1.01) |
| BMI | 1.07 (1.04-1.11) | 1.03 (1.00-1.07) | 1.03 (0.96-1.10) |
| ∆BMI | 1.00 (0.92-1.08) | 0.98 (0.90-1.07) | 1.02 (0.92-1.13) |
| ***All-cause mortality*** (12,495 participants, 688 events) | | | |
| WC | 1.01 (1.01-1.02) | 1.01 (1.00-1.01) | 1.00 (0.98-1.01) |
| ∆WC | 1.00 (0.99-1.02) | 1.00 (0.98-1.01) | 0.99 (0.97-1.00) |
| BMI | 1.05 (1.02-1.07) | 1.02 (1.00-1.05) | 1.03 (0.98-1.08) |
| ∆BMI | 1.05 (0.99-1.12) | 1.04 (0.98-1.12) | 1.08 (1.00-1.17) |
| Analyses were restricted to male participants with complete information on all adjusted variables. HRs are presented per 1 unit of measure: 1 kg/m^2^ higher BMI, and 1 cm higher WC). ∆PSM (population specific model) = baseline and change values for age, sex, systolic blood pressure, treated systolic blood pressure, total cholesterol, HDL cholesterol, smoking, diabetes. BMI, body mass index; CVD, cardiovascular disease; WC, waist circumference.  + Indicates the inclusion of either WC and change in WC or BMI and ∆BMI in the model  ++ indicates the inclusion of WC, change in WC, BMI and ∆BMI in the model | | | |
