# Supplementary material for: Waist circumference does not improve established cardiovascular disease risk prediction modeling
Source: PLoS One. 2020 Oct 2;15(10):e0240214. doi: 10.1371/journal.pone.0240214 (PMC7531816; doi:10.1371/journal.pone.0240214)
Supplement: S3 Table — (DOCX) [file pone.0240214.s003.docx]

| **S3 Table. Changes in model performance with the addition of WC and BMI: 10-year data** | | | | | |
| --- | --- | --- | --- | --- | --- |
|  | C-statistic | ∆ C-statistic | Likelihood Ratio *X*^2^ | AIC | HL |
| ***Fatal CVD events*** (22,915 participants, 198 events) | | | | | |
| FRS | 0.837 (0.811-0.864) | / | 303.1 | 1976.7 | 41.16 * |
| FRS + WC | 0.832 (0.805-0.860) | -0.005 | 307.5 | 1974.3 | 24.66 * |
| FRS + BMI | 0.837 (0.810-0.863) | 0.000 | 304.8 | 1977.0 | 28.04 * |
| FRS + WC + BMI | 0.829 (0.801-0.858) | -0.008 | 308.4 | 1975.4 | 25.41 * |
| PSM | 0.842 (0.816-0.864) | / | 374.7 | 1917.1 | 6.18 |
| PSM + WC | 0.843 (0.817-0.869) | 0.001 | 377.7 | 1916.1 | 3.90 |
| PSM + BMI | 0.843 (0.817-0.869) | 0.001 | 376.1 | 1917.7 | 3.68 |
| PSM + WC + BMI | 0.842 (0.816-0.869) | 0.000 | 378.0 | 1917.8 | 4.62 |
| ***Non-fatal CVD events*** (14,885 participants, 372 events) | | | | | |
| FRS | 0.775 (0.752-0.797) | / | 270.6 | 3212.8 | 71.06 * |
| FRS + WC | 0.775 (0.752-0.791) | 0.000 | 270.6 | 3214.8 | 69.41 * |
| FRS + BMI | 0.774 (0.752-0.797) | -0.001 | 271.0 | 3214.4 | 66.15 * |
| FRS + WC + BMI | 0.775 (0.752-0.797) | 0.000 | 271.5 | 3215.9 | 64.68 * |
| PSM | 0.781 (0.758-0.803) | / | 391.3 | 3104.1 | 16.17 * |
| PSM + WC | 0.781 (0.759-0.804) | 0.000 | 391.9 | 3105.5 | 17.67 * |
| PSM + BMI | 0.781 (0.758-0.803) | 0.000 | 391.3 | 3106.1 | 15.46 |
| PSM + WC + BMI | 0.782 (0.759-0.804) | 0.001 | 394.3 | 3106.1 | 14.93 |
| ***All-cause mortality*** (22,915 participants, 534 events) | | | | | |
| FRS | 0.772 (0.750-0.794) | / | 515.4 | 4558.8 | 70.64 * |
| FRS + WC | 0.770 (0.748-0.794) | -0.002 | 516.5 | 4559.7 | 60.51 * |
| FRS + BMI | 0.773 (0.751-0.794) | 0.001 | 515.5 | 4560.7 | 71.18 * |
| FRS + WC + BMI | 0.770 (0.747-0.791) | -0.002 | 522.3 | 4555.9 | 56.93 * |
| PSM | 0.781 (0.760-0.802) | / | 624.1 | 4462.1 | 24.38 * |
| PSM + WC | 0.781 (0.760-0.802) | 0.000 | 626.6 | 4461.6 | 27.56 * |
| PSM + BMI | 0.781 (0.760-0.802) | 0.000 | 624.5 | 4463.7 | 26.13 * |
| PSM + WC + BMI | 0.781 (0.761-0.802) | 0.000 | 628.3 | 4461.9 | 24.55 * |
| Analyses were restricted to male participants with complete information on all adjusted variables who had baseline measures at least 10 years before December 31, 1993. FRS (Framingham Risk Score) as published by D’Agostino et al., 2008(27). PSM (population specific model) = age, sex, systolic blood pressure, treated systolic blood pressure, total cholesterol, HDL cholesterol, smoking, diabetes. BMI, body mass index; WC, waist circumference.  * Indicates significance (*P*<.05) | | | | | |
